# Remarks on Life Feasibility on the Red Planet

**DOI:** 10.3390/microorganisms13051105

**Published:** 2025-05-11

**Authors:** Fiorella Mancini, Giuseppe Graziano

**Affiliations:** Dipartimento di Scienze e Tecnologie, Università del Sannio, Via Francesco de Sanctis snc, 82100 Benevento, Italy; fmancini@unisannio.it

**Keywords:** Mars, liquid water, life, salty brines, perchlorates, protein stability, perchlorate tolerance

## Abstract

The current strong interest in the exploration of Mars leads to the question of the actual possibility of the presence or possible past or future development of life on the planet. Several clues suggest that liquid water could be stably present under the surface of Mars, but on the condition that it is rich in perchlorate salts, abundant in the Martian soil, which would allow for water to remain liquid at the very low temperatures found on the planet. In this work, the main evidence on the permissiveness of Martian environments to microbial life is reviewed, with particular attention to the evaluation of the tolerance limit to the perchlorates of different microorganisms. Furthermore, a reasonable theoretical approach is offered to calculate the stability of globular proteins in aqueous solutions rich in perchlorates, trying to provide, given the current lack of valid experimental data, a rational means to try to understand the behaviour of proteins in environmental conditions very far from those of Earth.

## 1. Introduction

The extraordinary technological developments that this millennium is experiencing have enormously changed every aspect of our lives, from those closest to us, such as telecommunications or transport, to those more distant, at least apparently, such as space exploration. The conquest of space has made Mars the alien planet of the solar system that we know best, although there are still many unknowns relating to this celestial body, starting from the possibility of the presence of life on the planet, or at least its habitability.

Thanks to spacecrafts launched into orbit around the planet and rovers sent to explore its surface and atmosphere, it is well known that the Red Planet has very different geological and climatic features from those of Earth, which make it decidedly less hospitable to life than Earth, at least as we know it. To date, there is no proof of the existence of Martian life. Nevertheless, it might be worth asking whether this possibility should be excluded *tout court*, also considering the past geological eras of the planet and the actual possibilities of future human exploration/colonization.

Observations of Mars’ surface have revealed environments characterized by high salinity, very low temperatures, presence of a low-pressure oxidative atmosphere, and high solar radiation due to the lack of a shielding layer of ozone [1,2]. For those reasons, the planet appears to be a very hostile place for life as it exists on our planet. However, it is necessary to consider that Earth also has very inhospitable environments, which, although considered uninhabitable in the past, have proven to be teeming with life. In fact, environments on Earth characterized by extreme values of pressure, pH or temperature, very high salinity, or exposure to powerful solar radiation are surprisingly inhabited by various microorganisms, collectively referred to as extremophiles, as they have adapted to live in conditions considered extreme for more “ordinary” living species, including humans. For example, although the deep-sea brines of the Red Sea present a combination of extreme environmental conditions, such as high salinity, the presence of heavy metal, high hydrostatic pressure, and lack of oxygen, they thrive on life in the form of bacteria [3,4,5,6,7], archaea [8], and viruses [9]. Likewise, the harsh environmental conditions on Mars do not automatically exclude the possibility of the presence of adapted life forms. The aim of this work is to highlight what could be the main critical issues for the development of life on the Red Planet and try to evaluate whether they can be overcome and to what extent.

## 2. Water on Mars

### 2.1. Evidence of Liquid Water on Mars

In any environment, whether terrestrial or extra-terrestrial, the search for life always begins as a search for water. Water has unique chemical properties that make it the ideal solvent for the occurrence of the biological processes and chemical reactions that take place within cells and that are essential for life. For this reason, in the debate on the possibility of the existence, past or present, or potential future development of life on the Red Planet, many efforts have been devoted and still are devoted in investigating the presence of water on the planet.

**Figure 1:**
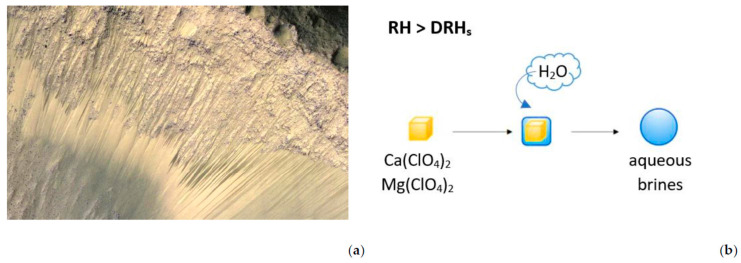
Recurring slope lineae on Mars’s surface. (**a**) RSL at a crater on the floor of Valles Marineris, near the Martian equator, captured by the HiRISE (High Resolution Imaging Science Experiment). NASA/JPL-Caltech/Univ. of Arizona. (**b**) Deliquescence process of perchlorates. On Mars, salts such as calcium perchlorate [Ca(ClO_4_)_2_] and magnesium perchlorate [Mg(ClO_4_)_2_] may be able to absorb water vapour from the atmosphere and dissolve into it to form aqueous brines. This process requires that relative humidity (RH) be higher than the deliquescence relative humidity of each salt (DRHs).

Geomorphological and mineralogical evidence shows how, during past geological eras, milder climatic conditions allowed for the stable presence of large amounts of liquid water on the planet’s surface [10,11,12,13,14,15,16]. However, the current conditions on the surface of Mars, characterized by low temperatures (average temperature 210 K/−63 °C [17]) and low atmospheric pressure (average pressure about 610 Pa [18]), now make the stable presence of liquid water impossible. Today, water on Mars’ surface is mostly found either in frozen form, particularly in the northern polar cap and beneath a permanent ice cap of carbon dioxide at the south pole [16,19,20,21,22,23], or as modest amounts of water vapour in the atmosphere [24,25,26]. However, the first observations of the so-called Recurring Slope Lineae (RSL) (Figure 1) have opened up the possibility of an at least temporary presence of liquid water on Mars’ surface [27,28]. RSL are long dark streaks, between 0.5 and 5 m wide, which appear with recurrence on Mars’ surface. They usually start from the top of rocky slopes and gradually extend during the warmer Martian seasons, while equally gradually disappearing during the colder seasons, following an annual cycle [27].

### 2.2. Martian Brine: Formation and Composition

The origin and nature of RSL are subjects of debate. While some believe that they are rivulets of aqueous brines formed by deliquescence of the salts present in the Martian soil [29,30,31], others suggest they are dry flows of granular material, such as sand or debris, fed by the seasonal behaviour of the winds [32,33]. The hypothesis of aqueous brines is supported by the abundant presence of hygroscopic salts in the Martian soil, which include various compounds of chlorine [34,35,36,37,38,39,40,41,42,43,44,45]. In particular, analyses of Martian soil samples revealed the presence of different types of sulphates (SO_4_^2−^) [42,46,47,48,49], chlorides (Cl^−^) [29,30,50], chlorates (ClO_3_^−^) [50,51], and perchlorates (ClO_4_^−^) [35,51,52], with calcium perchlorate [Ca(ClO_4_)_2_] and magnesium perchlorate [Mg(ClO_4_)_2_] being among the most abundant [36,39]. These salts are capable of absorbing water present in the atmosphere in vapour form, forming a liquid solution in which they dissolve through a process known as deliquescence. The lowering of the freezing temperature of the resulting saline solution (known as the eutectic temperature) would allow for the presence, albeit temporarily, of liquid water on the planet at temperatures, at which it would naturally be frozen [31,53]. The phenomenon of deliquescence occurs at a certain relative humidity (RH), known as deliquescence relative humidity (DRH), which is characteristic for each salt. On Earth, the deliquescence process has been observed in the driest terrestrial environment, the Atacama Desert (Chile), where adequate RH conditions allow for the sodium chloride (NaCl) present in the halite crusts to absorb water from the atmosphere and form brines that are permissive to the survival of photosynthetic and heterotrophic microorganisms [54,55,56,57]. An interesting model of the climatic conditions on Mars, including RH and temperature, showed that a Cl^−^ deposit region on Mars reaches the DRH values of three different hygroscopic salts (NaCl, calcium chloride (CaCl_2_) and magnesium chloride (MgCl_2_)) several times over the course of a Martian year [53]. However, other factors, such as the density, porosity, and specific composition of the Martian soil, which are difficult to predict and, therefore, reproduce in the laboratory, can influence the actual occurrence of the deliquescence process, as well as the quantity and stability of any resulting brine [53]. In any case, for the saline mixture to have a eutectic temperature lower than the temperatures of the Martian surface, a high concentration of salts is required, especially perchlorates, as they are known for their ability to significantly reduce the freezing point of water.

To assess the permissibility to life in Martian salty brines, it is necessary to investigate what realistic concentrations of potential perchlorate solutions could be, and what features potential microorganisms should have to resist in such environments. The quantification of perchlorate concentrations, carried out in the Wet Chemistry Lab of the Phoenix lander by adding approximately 1 cm^3^ of Martian soil to 25 mL of water, returned values between 2.2 and 2.5 mM. However, these concentrations are far below those of the aqueous perchlorate mixtures realistically expected on Mars. For example, for sodium perchlorate (NaClO_4_), at its eutectic temperature (239 K/−34 °C), an aqueous solution with a concentration of around 6.2 M has been estimated [29]. Similar conditions make halophilic microorganisms an interesting model to evaluate the possibility of the existence of life on the Red Planet. However, as anticipated, beyond the scarcity of water and low temperatures, the surface of Mars remains very inhospitable due to exposure to powerful solar radiation and oxidizing atmosphere. Thus, greater possibilities for the presence of life on Mars lie in the environments beneath the planet’s surface.

### 2.3. Subglacial Liquid Water on Mars

In July 2018, the Italian Space Agency provided evidence of the existence under the southern polar ice cap of Mars of a subglacial lake 1.5 km deep and approximately 20 km wide [58]. The presence of the lake was inferred from radar measurements taken by the MARSIS radar on board of the European Mars Express orbiter. Its existence is actually debated because other factors, such as an adequate amount of geothermal heating flow coming from the crust surface and pressure of the ice above, would be required to support brine formation [59]. On the other hand, factors such as a polar ice cap flow are hypothesized to facilitate subglacial water accumulation [60]. Two years later, the first evidence of a subglacial lake on Mars, three other lakes of more modest dimensions, were detected near the previous one in a similar way [61]. According to the main hypothesis, all water stored under the surface of Mars could be the residue of water that was once found on the planet’s surface.

On Earth, similar radar measurements have detected hundreds of subglacial lakes beneath the Antarctic Ice Sheet, the Greenland Ice Sheet [62], and Iceland’s Vatnajökull ice cap. The peculiar characteristics of these environmental niches, which make them permissive to the life of certain extremophilic species [63], has opened up the possibility of the presence of life also in the subglacial lakes on Mars, which could represent the habitat of microorganisms similar to those present under the ice sheets on Earth. However, even for the hypothesis of the presence of subglacial lakes, the low temperatures estimated under the southern polar ice cap of Mars (205 K/−68 °C) suggest that any underlying liquid water present is found in the form of briny pools rich in perchlorate salts deriving from the Martian soil [58]. So, if any microorganism inhabits or has inhabited these salty brines, it must have developed the ability to live at very high perchlorate concentrations.

## 3. Perchlorate Salts

### 3.1. Detection of Perchlorates on Mars

Whether we decide to consider the surface of the Red Planet or its subsurface, if water is present in liquid form somewhere on the planet, it must be rich in hygroscopic salts, especially perchlorates. The presence of perchlorate salts in the Mars’ soil is supported by various evidence, including in situ detections during Mars exploration missions, such as Phoenix [35,38,64], Curiosity [37,39,40,41], and Perseverance [42,43,44,45,65], and analyses of meteorites from Mars [36,66]. Several species of perchlorate have been detected on Mars, of which Mg(ClO_4_)_2_ and Ca(ClO_4_)_2_ appear to be the most abundant [36,67]. NaClO_4_ has also been detected in the Martian soil [42,45,65], and, although less common, other types of perchlorate may also be present [67].

Analyses of rock samples from the Curiosity and Phoenix landers revealed that perchlorate levels can vary significantly, even between extremely close regions and in morphologically similar samples. In other cases, instead, regions very distant from each other showed similar concentrations of perchlorates [37,39,40,41]. The distribution of perchlorates at different depths is less understood, but shallow samples from the Phoenix lander indicated consistent perchlorate levels in the surface and subsurface layers [35], suggesting efficient mixing on this scale [64].

The origin of perchlorates on Mars is a matter of debate, starting from the primary site of formation of these salts. While according to some hypotheses, the synthesis of perchlorates would take place in the atmosphere, according to others, it would take place directly in the Martian soil. Thus, several mechanisms have been proposed, each based on interactions between chlorine compounds and environmental factors [68,69,70]. On Earth, perchlorates are formed primarily in the upper atmosphere through the photochemical oxidation of chlorine by the ozone. However, on Mars, given its thinner atmosphere and the presence of different oxidizing agents, it might be reasonable to think that the production of perchlorates occurs through the combination of several proposed mechanisms rather than a dominant pathway.

### 3.2. Chemical Properties of Perchlorates

Perchlorates have characteristic chemical properties that define their behaviour and reactivity [71]. Under standard conditions, perchlorates are kinetically stable, being slow to decompose and, therefore, persisting for long periods in the environment. However, they also are strong oxidizers and, as such, can be reactive, especially under certain conditions, where they can decompose and release oxygen. This strong oxidizing nature has implications for both the potential generation of oxygen and their toxicity to organisms. Furthermore, they are highly soluble and dissolve easily in water. These properties give them potential environmental mobility and high bioavailability in aqueous systems. On Mars, this behaviour could help explain their distribution in surface and underground soils. As anticipated, perchlorates are very deliquescent, meaning that they can absorb water from the atmosphere and form a liquid solution in which they dissolve, and have low eutectic temperatures, which allow them to form liquid solutions at much lower temperatures compared to many other salts.

**Figure 2:**
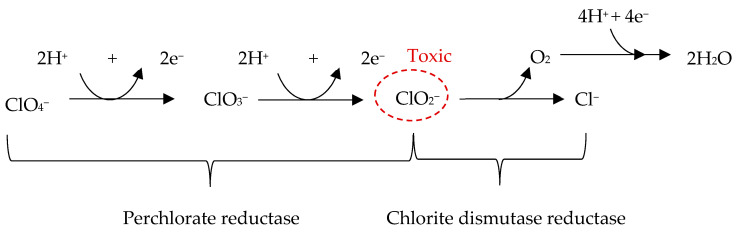
Pathway for the ClO_4_^−^ reduction to Cl^−^ via intermediate steps. The reaction starts with the reduction of ClO_4_^−^ to ClO_3_^−^ by perchlorate reductase, with the release of a water molecule. ClO_3_^−^ is further reduced to ClO_2_^−^ by the same enzyme, perchlorate reductase, producing another molecule of water. ClO_2_^−^, which has toxic potential, is broken down into Cl^−^ and O_2_ by the enzyme chlorite dismutase. Finally, the O_2_ produced can undergo reduction by the addition of four hydrogens to form two more water molecules.

An interesting property of perchlorates is their chaotropicity, which is the ability of some compounds to disrupt the ordered structure of water and other solvents [72,73,74], and to alter the stability of macromolecules such as proteins, DNA, and cell membranes [75]. Chaotropic agents destabilize the non-covalent interactions that typically stabilize biomolecular structures, making them more prone to denaturation. Because of this effect, perchlorates may alter biological processes and interfere with cellular structure and function, resulting in toxicity to many life forms, although specific studies on the effects of perchlorates on biological macromolecules are lacking. There are few studies assessing the toxicity of perchlorates for humans. They appear to be dangerous at high doses, and this represent an obstacle to potential future human exploration of the Red Planet. Furthermore, ionizing radiation on Mars could cause the breakdown of perchlorate into other chlorine oxyanions such as chlorite (ClO_2_^−^) and hypochlorite (ClO^−^), which, being more reactive, may pose additional toxic potential [76]. For example, it has been shown that perchlorates in the presence of radiation increase the decomposition of amino acids [77]. For these reasons, microorganisms capable of decomposing perchlorates could be useful to reduce their presence in the Martian regolith, reclaiming it before potential in situ human applications [78]. Perchlorate- and chlorate-reducing bacteria are widely distributed on Earth [79]. They include some halotolerant species, such as *Serratia marcescens Bizio 1823* [80], and exhibit variable metabolic and morphological features [79,81]. Most of these bacteria grow anaerobically by reducing ClO_4_^−^ and ClO_3_^−^ to Cl^−^. For example, the Beta-proteobacteria genera *Dechloromonas* and *Azospira* are capable of completely oxidizing organic carbon or various inorganic electron donors, such as H_2_, H_2_S, or Fe^2^⁺, using perchlorate as an electron acceptor under anoxic conditions [82]. Two key enzymes with perchlorate-reducing activity have been identified in some marine bacteria: perchlorate reductase and chlorite dismutase [83,84]. The former catalyzes the reduction of ClO_4_^−^ to ClO_2_^−^, which is then converted into Cl^−^ and molecular oxygen (O_2_) by chlorite dismutase [81] (Figure 2).

## 4. Perchlorate Salts and Proteins

### Effects of Perchlorate Salts on Protein Stability and Protein–Solvent Interactions

As chaotropic agents, perchlorates destabilize protein structures, although specific and systematic measurements of the effects of these compounds on the various classes of biological macromolecules are scarce. It would be important to study the interactions between proteins and perchlorates, given the fundamental and multifaceted role of these macromolecules in the forge of life. The broad spectrum of functions that proteins perform in cells (structural, enzymatic, signalling, transport, etc.) gives them unique characteristics among biological macromolecules. To properly cope with multiple tasks, proteins occur in different structures (globular, fibrous, protein membrane, or disordered), which provide them with the support necessary for their function. Although understanding the behaviour of proteins in solutions rich in hygroscopic salts, such as perchlorates, would be an important contribution to solving the enigma of the presence of life in extra-terrestrial environments, the necessary experimental measurements are very difficult to carry out in the laboratory due to the difficulty of reproducing the conditions of interest, primarily the low temperatures, well below 0 °C, which occur on Mars. Thus, the difficulty of having adequate laboratory equipment gives way to theoretical models to try to give a plausible point of view on the question of the behaviour and stability of proteins in aqueous solutions rich in perchlorates.

**Figure 3:**
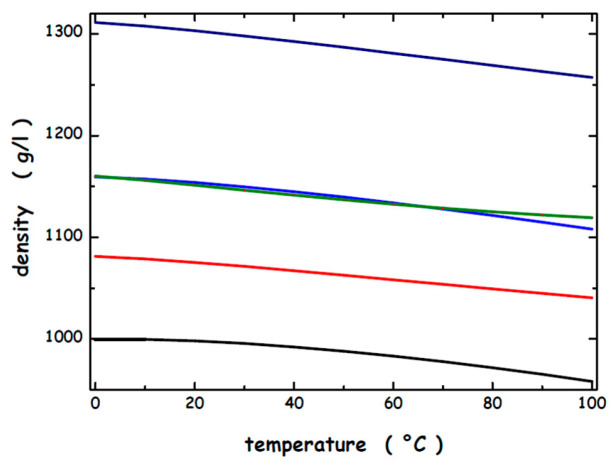
Experimental density of water (black curve), 1 M NaClO_4_ (red curve), 2 M NaClO_4_ (blue curve), 1 M Mg(ClO_4_)_2_ (green curve), and 2 M Mg(ClO_4_)_2_ (navy curve) over the 0–100 °C temperature range.

One of us devised a theoretical approach to model and rationalize the conformational stability of globular proteins [85,86,87,88]; full details can be found in the original articles. In the assumption that the polypeptide chains populate only two macrostates, the N-state and the D-state, the Gibbs free energy change associated with denaturation (i.e., the reversible passage from the N-state to the D-state) is given by the following:

ΔG_d_ = ΔΔG_c_ – T·ΔS_conf_ + ΔE_a_ (1)

where ΔΔG_c_ = ΔG_c_ (D-state) − ΔG_c_ (N-state) is the difference in the reversible work needed to create, in water or an aqueous solution, a cavity suitable to host the D-state or the N-state; ΔS_conf_ measures the gain in conformational entropy of the polypeptide chain on passing from the N-state to the D-state; ΔE_a_ = E_a_ (D-state—water) − E_a_ (N-state—water) + ΔE (intra-protein) represents the difference in energetic interactions between the two states. The ΔΔG_c_ term is always positive (i.e., it stabilizes the N-state) because the conformations belonging to the D-state have a water-accessible surface area [89,90], WASA, larger than that of the N-state, and so the creation of a cavity suitable for them is more costly [note the following: (a) experimental measurements indicate that the volume change associated with denaturation is negligibly small [91,92], and so it is correct to assume that the N-state and D-state have the same van der Waals volume, V_vdW_; (b) it has been demonstrated, via analytical relationships and computer simulations [93,94,95,96], that, even though the cavity V_vdW_ is kept fixed, the ΔG_c_ magnitude increases with cavity WASA; and (c) the shape of the cavity matters]. The geometric basis of the reversible work of cavity creation [93,97] allows for the use of simple objects to model the N-state and the D-state: a sphere for the N-state and a prolate spherocylinder for the D-state, possessing the same V_vdW_ but different WASA. In addition, it is possible to use the analytical relationships provided by classic scaled particle theory [97], SPT, to calculate ΔG_c_ in pure liquids and in solutions. To perform classic SPT calculations, it is necessary to fix several geometric measures. The model protein has 138 residues and V_vdW_ = 14,137 Å^3^; the N-state sphere has radius *a* = 15 Å and WASA = 3380 Å^2^; the D-state prolate spherocylinder has radius *a* = 6 Å, cylindrical length *l* = 117 Å, and WASA = 6128 Å^2^ (they are the same geometric models and values used in our previous studies). In addition, in classic SPT calculations, we use the experimental density of water and the chosen aqueous solutions over a large temperature range and assign reliable values to the effective hard sphere diameters of water molecules and ions [98,99]. The density values of water, 1 M and 2 M NaClO_4_ aqueous solutions, and 1 M and 2 M Mg(ClO_4_)_2_ aqueous solutions over the 0–100 °C temperature range are reported in Figure 3. It is evident that the addition of these perchlorate salts to water leads to a significant density increase, a consequence of the strength of ion–water energetic interactions. In this respect, it is worth noting that Dougan and colleagues performed neutron scattering measurements of Mg(ClO_4_)_2_ aqueous solutions at its eutectic concentration, 3.5 M, at a very low temperature, 216 K [100]. A dramatic effect on the water structure emerged with the complete collapse of the second peak toward the first peak of the oxygen–oxygen radial distribution function; it corresponds to the effect of about 2000 atm pressure. Moreover, there are six water molecules in the first coordination shell of magnesium ion, and probably more in the first coordination shell of perchlorate. Figure 3 also indicates that the addition of perchlorate salts to water causes a shift in the temperature of maximum density below 0 °C (such an effect is general [101,102]) and this renders unfeasible and unreliable an extrapolation of the density values to lower temperatures. This is a serious limit, considering that the temperature on Mars is well below 0 °C, and emphasizes the need to perform experimental density measurements.

The assigned diameters are as follows: σ(H_2_O) = 2.80 Å, corresponding to the location of the first peak in the oxygen–oxygen radial distribution function of water, as determined by means of X-ray and neutron scattering measurements [103,104]; σ(Na^+^) = 2.02 Å, σ(Mg^++^) = 1.44 Å, and σ(ClO_4_^−^) = 4.80 Å, corresponding to the Pauling-type ionic diameters [105].

**Figure 4:**
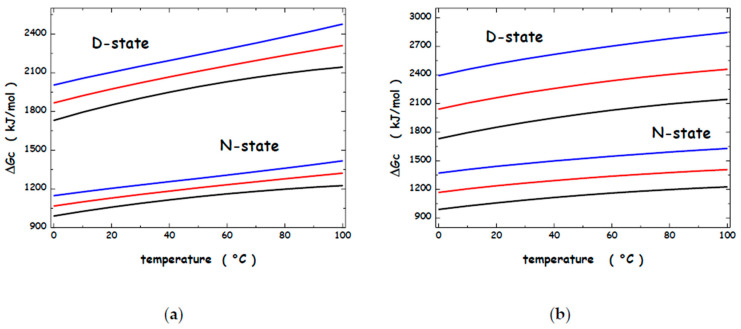
Temperature dependence of the ΔGc functions for the sphere representing the N-state and the prolate spherocylinder representing the D-state in water (black curves), 1 M NaClO_4_ (red curves), and 2 M NaClO_4_ (blue curves) aqueous solutions (**a**) and in water (black curves), 1 M Mg(ClO_4_)_2_ (red curves), and 2 M Mg(ClO_4_)_2_ (blue curves) aqueous solutions (**b**).

The ΔG_c_ values for the N-state and the D-state of the model protein have been calculated over the 0–100 °C temperature range in water, 1 M and 2 M NaClO_4_ aqueous solutions, and 1 M and 2 M Mg(ClO_4_)_2_ aqueous solutions, and are shown in Figure 4a and 4b, respectively. The reported curves indicate that ΔG_c_(H_2_O) < ΔG_c_(1 M NaClO_4_) < ΔG_c_(2 M NaClO_4_) < ΔG_c_ [1 M Mg(ClO_4_)_2_] < ΔG_c_[2 M Mg(ClO_4_)_2_] over the whole considered temperature range. This ranking is due to the coupling between the density increase caused by the perchlorate salt addition to water (see Figure 3) and the increase in the volume-packing density—the volume fraction really occupied by molecules and ions (look at the numbers listed in the third column of Table 1). These results indicate that the ΔΔG_c_ contribution rises on adding perchlorate salts to water, stabilizing the N-state. However, NaClO_4_ and Mg(ClO_4_)_2_ have a destabilizing action toward the N-state of globular proteins: experimental data indicate, for instance, that the denaturation temperature of RNase A decreases by 10 °C in 1 M NaClO_4_ [106], that of α-chymotrypsin decreases by 15 °C in 1 M NaClO_4_, and by 20 °C in 0.5 M Mg(ClO_4_)_2_ [107,108]. The measured destabilizing action should imply that the perchlorate ion is able to preferentially interact with protein surfaces and to favour the D-state that has a larger WASA. In the framework of our theoretical approach, these energetic interactions should lead to a large negative ΔE_a_ term, stabilizing the D-state. It is possible to make simple calculations for our model protein to gain perspective. It has been assumed that ΔE_a_ ≈ 0 in water because a polypeptide chain is able to do the same energetic interactions either folded or unfolded, taking into account those with water molecules and the intramolecular ones. Such a balance is lost in the presence of ions or molecules that have preferential energetic interactions with protein surfaces [109,110,111].

The ΔΔG_c_ values in H_2_O, 1 M and 2 M NaClO_4_, and 1 M and 2 M Mg(ClO_4_)_2_, calculated at 20 °C and 1 atm, are listed in the fourth column of Table 1; the T ΔS_conf_ term is calculated assuming an equal and independent contribution for each residue, 19 J/K mol per residue, both in water and aqueous solutions (i.e., it has the same magnitude in all the considered cases; see the sixth column of Table 1). The ΔG_d_ value in water at 20 °C and 1 atm is calculated via Equation (1) and is reported in Table 1. The ΔG_d_ values in the aqueous solutions of NaClO_4_ and Mg(ClO_4_)_2_ can solely be guessed on the basis of the destabilizing action of these salts; the fixed values are listed in the fifth column of Table 1.

The ΔE_a_ values obtained via Equation (1), listed in the seventh column of Table 1, indicate that, at 20 °C and 1 atm, the D-state is favoured in 1 M and 2 M Mg(ClO_4_)_2_ because the ΔE_a_ estimates are very large and negative. The perchlorate ion, with its tetrahedral structure, interacts well with water molecules, but, due to its large polarizability (it has a small charge density value because of its size), makes very good interactions with the groups on protein surfaces (note that there is not a large difference in chemical nature between exposed and buried surfaces of globular proteins) [112,113]. The perchlorate action should be similar to that of the thiocyanate ion [114].

## 5. Perchlorate Tolerance of Microorganisms

### 5.1. Evidence and Implications of Perchlorate Resistance of Terrestrial Microorganisms for Putative Life on Mars

Among the microorganisms investigated so far, heterotrophic prokaryotes and eukaryotes show the greatest tolerance to perchlorates. The most tolerant prokaryote detected so far is *Planococcus halocryophilus*, a halotolerant bacterium isolated from permafrost in the Canadian High Arctic [115]. This microorganism, when exposed to gradually increased concentrations of perchlorates, is capable of tolerating up to about 1.0 M of Mg(ClO_4_)_2_ and about 0.1 M of Ca(ClO_4_)_2_, forming large cell clusters presumably as a response to stress caused by the chaotropicity of perchlorate [116]. Other prokaryotes, although adapted to high concentrations of salts, show limited tolerance to perchlorate, with the archaea *Haloferax volcanii* and *Halorubrum lacusprofundi* tolerating up to 0.6 M [117] and 0.8 M [118] NaClO_4_, respectively. The limited perchlorate tolerance in these species may be due to additional stress resulting from the chaotropic and oxidative effects of perchlorate. Surprisingly, some eukaryotic microorganisms can grow at higher perchlorate concentrations than prokaryotic ones: the fungus *Purpureocillium lilacinum* and the yeast *Debaryomyces hansenii* tolerate about 1.9 M [119] and about 2.4 M [119] of NaClO_4_, respectively. Furthermore, *D. hansenii* can also withstand high levels of Mg(ClO_4_)_2_ (0.9 M) and Ca(ClO_4_)_2_ (0.5 M) [120].

*D. hansenii* is a halotolerant, osmotolerant, and xerotolerant (organisms capable of growing in conditions of low water activity (a_w_), a parameter measuring the availability of water for biochemical reactions and microbial growth in a substance, ranging from 0 for completely dry to 1.0 for pure water) microorganism which lives in salt-rich environments such as the salt flats of the Atlantic coast of Namibia and the Great Salt Lake in Utah, but it is also found as a contaminant in some foods [121]. Due to its ability to grow in habitats with low a_w_ and to survive salinity levels up to 25%, the yeast is used as a model organism in studies on salt toxicity [121]. Its capacity to tolerate even elevated levels of perchlorates makes it an interesting model to investigate the mechanisms responsible for this resistance. Some of the strategies used by resistant microorganisms to counteract chaotropic stress include the positive regulation of genes involved in protein stabilization and synthesis, lipid and energy metabolism, and membrane structure [122]. Similar responses could explain the ability of *D. hansenii* to resist chaotropic solutes. An investigation evaluating the resilience of *D. hansenii* under high-perchlorate conditions revealed the increased perchlorate-specific expression of stress-related proteins, which would help counteract oxidative stress and protect cellular components from the damage caused by perchlorates [123]. The study found that several key stress response pathways were activated, including processes such as protein glycosylation and cell wall remodulation. These results showed that *D. hansenii* responds to perchlorate-induced stress by improving its protective and reparative mechanisms. Furthermore, as a mechanism to counteract osmotic stress, halophilic and halotolerant microorganisms produce and accumulate compounds called compatible solutes. In particular, *D. hansenii* accumulates glycerol in response to salt stress [121]. One study showed that this compound has the ability to protect and renature a yeast inorganic pyrophosphatase exposed to temperatures up to 95 °C [124], while in the ascomycete *Aspergillus nidulans*, the ability to produce glycerol and erythritol as compatible solutes protected this microorganism from the effects of ethanol, a chaotropic compound that would act by altering water–macromolecule interactions [125]. Considering these pieces of evidence, it could be hypothesized that glycerol could contribute, in combination with other more specific adaptative mechanisms, to the resistance to perchlorates of *D. hansenii* by counteracting their destabilizing effects and supporting protein stability. It is clear that specific studies would be needed to validate this hypothesis. However, the observed proteomic response of *D. hansenii* raises the possibility that similar microorganisms may be able to resist Martian-like brines by implementing specific stress response mechanisms and that, *a fortiori*, potential microorganisms which have evolved and adapted to live in Martian environments may have developed the ability to survive in brines with high concentrations of perchlorate.

### 5.2. Effects of Perchlorate on Microorganisms Exposed to Low Temperatures

As already mentioned, chaotropic compounds generally have destabilizing effects on the structure of macromolecules, causing stress to cells. On the other hand, low temperatures tend to favour intermolecular non-covalent interactions and therefore can stiffen biological macromolecules and cell membranes in cells, altering their functions. Thus, it has been hypothesized that the destabilizing effect of chaotropic agents can counteract molecular rigidity due to low temperatures, increasing the ability to survive at low temperatures of some extremophilic microorganisms [126]. In fact, it was observed that, unlike kosmotropic substances, some chaotropic compounds expanded the life window of some species of xerophilic fungi at low temperatures. Furthermore, the tested fungi accumulated preferably chaotropic rather than kosmotropic metabolites in the cytoplasm, presumably to counteract the osmotic effect and/or molecular stiffening induced by low temperatures [126]. In other words, chaotropic agents, by altering the structure of water bulk and reducing molecular interactions, can decrease the minimum temperature at which cell division can occur by increasing the growth rate at low temperatures. These effects could be due to an increase in the flexibility of macromolecules attributable to chaotropic agents. In support of this hypothesis, it has been observed that some microorganisms shift their optimal salinity conditions for growth towards higher concentrations of salts when exposed to low temperatures [127].

However, even the low temperature that would characterize the Martian brines would represent a challenge for the viability of any form of life due to the onset of physical processes such as intracellular vitrification. In fact, when cells are exposed to low temperatures, the freezing of extracellular water causes an increase in the concentration of extracellular solutes, which attract water from the intracellular environment, determining an increase in cellular viscosity to the point that the cell undergoes a glass transition (or intracellular vitrification), at which biochemical reactions stop [128]. In some microorganisms, it has been observed that this process occurs around −20 °C [129]. Nevertheless, a recent study showed that molar concentrations of Mg(ClO_4_)_2_ (2.5 M) lower the temperature peak of intracellular vitrification of *Bacillus subtilis* from −21.2 °C to −83.2 °C [130], although this microorganism showed a tolerance limit to Mg(ClO_4_)_2_ of 0.3 M [131]. Thus, even in the case of the low temperatures of the possible Martian aqueous deposits, the presence of Mg(ClO_4_)_2_ at high concentrations could protect cells from intracellular vitrification in synergy with the development of adaptive cellular mechanisms. However, understanding whether, in addition to protecting cells from vitrification, perchlorate salts are also capable of extending the window of cellular metabolic processes at low temperatures remains a crucial point in the investigation of the feasibility of the development of life on the Red Planet.

Freeze/thaw experiments carried out on microorganisms may be useful for testing their ability to survive the strong temperature changes that occur on Mars [1]. Such temperature fluctuations can be stressful for cells, as the formation and melting of ice crystals can cause physical damage to cell membranes, osmotic stress, and protein denaturation. However, it is possible that, in high-salt brines, this damage is mitigated to some extent. It was observed that the presence of NaCl increased the survival capacity of *P. halocryophilus* during freeze/thaw cycles [132]. In particular, the salts lowered the freezing point of water, reducing the formation of harmful ice crystals and creating a more stable liquid environment for cells, even at subzero temperatures. As a result, some microorganisms were able to withstand repeated freeze/thaw cycles better in brines than in pure water. In the same study, it was also observed that the protective effect of the tested salts helped prevent dehydration, allowing for cells to retain their structural integrity across temperature cycles. Along the same lines, it has been found that, in deliquescent NaCl solutions, the a_w_ increases as the temperature decreases due to the precipitation of the salts [53]. Therefore, some salt-rich aqueous environments may become more habitable in terms of a_w_ as the temperature drops.

### 5.3. Kosmotropic Agents Can Protect Microorganisms from Chaotropic Perchlorate Stress

A study investigating microbial life in the seawater–brine interface of the brine Lake Kryos, located in the deep sea of the Mediterranean Ridge, showed that specialized archaea and bacteria have adapted to live in conditions of high salinity and chaotropic stress [133]. These microbial communities, exposed to chaotropic compounds, such as MgCl_2_, remained metabolically active despite the existence of conditions that exceeded the usual chaotropic limit for life. The concomitant presence in Lake Kryos of kosmotropic agents, such as sodium and sulphate ions, appeared to mitigate chaotropic stress by stabilizing proteins and cellular structures. Kosmotropic substances should strengthen hydrogen bonds between water molecules by promoting the formation and stability of hydration shells around biomolecules, thus helping to prevent their denaturation and maintain their integrity, even in high-salinity or chaotropic conditions. Therefore, in the salty environment of Lake Kryos, any kosmotropic substance present in the cytoplasm of the cells of the resident microbial communities or in the surrounding environment would help these microorganisms maintain membrane integrity, prevent protein denaturation, and stabilize intracellular processes. In other words, kosmotropic agents would indirectly contribute to microbial survival thanks to the counterbalance of the destabilizing effects of chaotropic compounds. Thus, even in perchlorates-rich Martian brines, the potential concomitant presence of kosmotropic agents could mitigate the destabilizing effects of chaotropic salts, making the extreme Martian environments less hostile to the proliferation of life.

## 6. Conclusions

The growing interest in the exploration of Mars has brought to the forefront the evaluation of the possibility of the presence or possible development of life on the planet. Several lines of evidence indicate that it is possible to find liquid water in a stable form under the surface of Mars, but only in the form of aqueous brines. Taking into account the composition of the soil and the extremely low temperatures of the Red Planet, these brines are thought to be rich in perchlorate salts, particularly Ca(ClO_4_)_2_ and Mg(ClO_4_)_2_, whose deliquescence property would allow for water to remain liquid at low Martian temperatures (about −70 °C). At the same time, the chaotropic nature of perchlorates, i.e., their ability to destabilize biomolecular structures by promoting the denaturation of proteins and interfering with cell membranes and the structure of DNA, poses a major challenge to the development of life on the planet, at least as we know it. The paucity of experimental data, obtained at high concentrations of perchlorates and low temperatures, on the effects of these salts on biomolecules, such as proteins, makes it extremely difficult to predict their behaviour in aqueous solutions rich in perchlorates. In this work, a model has been proposed that offers a rational theoretical basis for the calculation of the conformational stability of globular proteins in the aforementioned conditions in an attempt to overcome the limitations of current experimental measurements. Some measurements carried out so far [134], performed in conditions of perchlorate concentrations and temperatures very far from those of Mars, certainly have the merit of having tried to delve into this swampy field of research, but, as they do not seriously push in the right direction, unfortunately do not give a real contribution to the investigation of the proteome stability of the microorganisms tested. It must be recognized that it is technically difficult to experimentally reproduce the environmental conditions present on the Red Planet, starting from the low temperatures, which require very advanced and not easily available instrumentation to achieve. Therefore, so far, the most fruitful experimental results seem to be those coming from the analysis of the capacity of resistance to perchlorates of various species of microorganisms, both prokaryotic and eukaryotic, which have allowed us to identify possible model organisms, such as *D. hansenii*, a halo- and xerotolerant yeast capable of activating response strategies to perchlorate stress. Such data give hope for the ability of life to adapt to extreme conditions. Finally, other interesting research has investigated the effects on microorganisms of the combination of perchlorates with low temperatures or with kosmotropic compounds, underlining the need to take into account, in the evaluation of the feasibility of life, a multiplicity of agents involved in complex ecosystems and, above all, very different from those in which we are used to operating.

## Figures and Tables

**Table 1:** Values of experimental water molar density and volume-packing density of H_2_O, 1 M and 2 M NaClO_4_, and 1 M and 2 M Mg(ClO_4_)_2_ aqueous solutions at 20 °C and 1 atm; classic SPT-ΔΔG_c_ values for the model protein; estimates of ΔG_d_ and T·ΔS_conf_ at 20 °C and 1 atm; estimates of the ΔE_a_ term obtained via Equation (1). See text for further details.

	[H_2_O] M	ξ_3_	ΔΔG_c_ kJ mol^−1^	ΔG_d_ kJ mol^−1^	T·ΔS_conf_ kJ mol^−1^	ΔE_a_ kJ mol^−1^
H_2_O	55.4	0.383	792.2	23.6	768.6	0
1 M NaClO_4_	52.9	0.404	844.5	10	768.6	−65.9
2 M NaClO_4_	50.3	0.423	900.0	5	768.6	−126.4
1 M Mg(ClO_4_)_2_	51.7	0.428	924.3	−10	768.6	−165.7
2 M Mg(ClO_4_)_2_	47.6	0.471	1075.2	−30	768.6	−276.6

## Data Availability

No new data were created or analyzed in this study. Data sharing is not applicable to this article.
